# On the Question of CO’s Ability to Induce HO-1 Expression in Cell Culture: A Comparative Study Using Different CO Sources

**DOI:** 10.1021/acschembio.3c00750

**Published:** 2024-02-10

**Authors:** Xiaoxiao Yang, Qiyue Mao, Binghe Wang

**Affiliations:** Department of Chemistry and Center for Diagnostics and Therapeutics, Georgia State University, Atlanta, Georgia 30303, United States

## Abstract

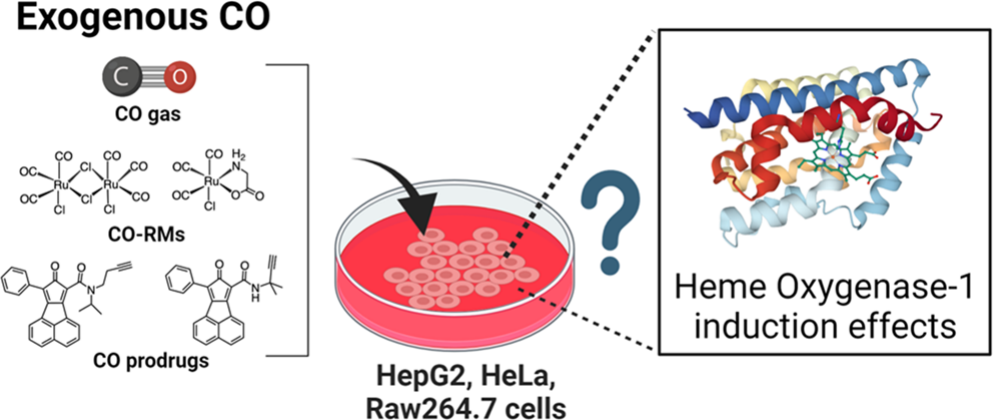

With the recognition of the endogenous signaling roles and pharmacological functions of carbon monoxide (CO), there is an increasing need to understand CO’s mechanism of actions. Along this line, chemical donors have been introduced as CO surrogates for ease of delivery, dosage control, and sometimes the ability to target. Among all of the donors, two ruthenium–carbonyl complexes, CORM-2 and -3, are arguably the most commonly used tools for about 20 years in studying the mechanism of actions of CO. Largely based on data using these two CORMs, there has been a widely accepted inference that the upregulation of heme oxygenase-1 (HO-1) expression is one of the key mechanisms for CO’s actions. However, recent years have seen reports of very pronounced chemical reactivities and CO-independent activities of these CORMs. We are interested in examining this question by conducting comparative studies using CO gas, CORM-2/-3, and organic CO donors in RAW264.7, HeLa, and HepG2 cell cultures. CORM-2 and CORM-3 treatment showed significant dose-dependent induction of HO-1 compared to “controls,” while incubation for 6 h with 250–500 ppm CO gas did not increase the HO-1 protein expression and mRNA transcription level. A further increase of the CO concentration to 5% did not lead to HO-1 expression either. Additionally, we demonstrate that CORM-2/-3 releases minimal amounts of CO under the experimental conditions. These results indicate that the HO-1 induction effects of CORM-2/-3 are not attributable to CO. We also assessed two organic CO prodrugs, BW-CO-103 and BW-CO-111. BW-CO-111 but not BW-CO-103 dose-dependently increased HO-1 levels in RAW264.7 and HeLa cells. We subsequently studied the mechanism of induction with an Nrf2-luciferase reporter assay, showing that the HO-1 induction activity is likely due to the activation of Nrf2 by the CO donors. Overall, CO alone is unable to induce HO-1 or activate Nrf2 under various conditions in vitro. As such, there is no evidence to support attributing the HO-1 induction effect of the CO donors such as CORM-2/-3 and BW-CO-111 in cell culture to CO. This comparative study demonstrates the critical need to consider possible CO-independent effects of a chemical CO donor before attributing the observed biological effects to CO. It is also important to note that such *in vitro* results cannot be directly extrapolated to *in vivo* studies because of the increased level of complexity and the likelihood of secondary and/or synergistic effects in the latter.

## Introduction

It was found in the 1940s that carbon monoxide (CO) is produced endogenously. In the 1960s, the major source of CO production was found to be through heme degradation by heme oxygenase (HO),^1−4^ which were later characterized to have two isoforms, inducible HO-1 and constitutive HO-2. Such endogenous production of CO implies a pathophysiological role for the HO–CO axis. Indeed, HO-1 has been known to be cytoprotective since the 1980s^5,6^ and used to be referred to as heat shock protein (Hsp) 32 because of its inducible nature by stress.^7^ The majority of the effects of heme degradation products at the time were attributed to the antioxidant power of the bile pigments.^8^ In 1991, intensive studies of CO’s pharmacological and signaling effects were first described,^9^ with the demonstration that exposure to CO at high concentrations activated soluble guanylyl cyclase *ex vivo*.^10^ The first report of the protective effects of inhaled CO was later described in animal models of lung injury.^11^ Then, the study of CO was pushed to the forefront.^12^ Inhaled CO at low, tolerable levels has been shown to modulate various physiological and pathological processes including inflammation^13^ and apoptosis^14,15^ and offer organ-protection activities.^16,17^

Most of the early works in the CO field were done by using CO gas. However, due to the intricate nature of setting up gas chamber experiments and concerns of lab personnel exposure to CO, there has been extensive interest in developing alternative delivery strategies. In the early 2000s, two ruthenium-based carbonyl complexes were introduced as “CO-releasing molecules (CORMs).” These are referred to as CORM-2 and CORM-3.^18,19^ Due to their commercial availability, these two CORMs have been widely used, appearing in >500 publications. In numerous cases, these CORMs were used as the only source of CO and the observed activities of these CORMs were attributed to CO through deductive reasoning. However, recent years have seen a large number of reports (>25 papers, e.g., refs (20−28)) showing very pronounced chemical reactivities and CO-independent biological activities of these CORMs, raising questions as to whether the observed biological activities can be attributed to CO. Readers are referred to a recent comprehensive review on this subject.^29^

In studying the HO-1–CO axis, there is a common inference that a key mechanism by which CO exerts its effects at the cell culture level is through the induction of HO-1 expression. If true, this would be a positive feedback loop as HO-1 is an enzyme that catalyzes the generation of CO. Positive feedback loops are not nearly as common as negative feedback loops and only exist for special physiological or pathological reasons such as signal amplification or mutation of negative regulator(s).^30−32^ Some commonly known examples include blood coagulation, NF-κB/K-Ras, and ER-α36/EGFR signaling pathways leading to oncogenesis, to name a few. In the case of HO-1 and CO, we became interested in the reported positive feedback loop for several reasons. First, if such a positive feedback loop does exist, it likely means that there is one (or more) unknown mechanism to prevent this loop from going into “perpetuity.” Levitt has raised the issue of whether there is a sufficient supply of heme for CO to play a signaling role in the same fashion as second messengers that need to increase concentrations quickly, such as in the case of cA(G)MP.^33^ Indeed, exhaustion of heme could serve as a “brake” for this otherwise exponential amplification process.^33^ Further, if heme availability is the likely “rate-limiting” step, then what is the reason for a positive feedback loop? Is this for the rapid elimination of toxic levels of heme under injurious conditions or is it because HO-1 may play other roles beyond catalyzing heme degradation?^34,35^ Second, the proposed central roles that HO-1 plays in CO biology mean that the purported HO-1 induction by exogenous CO in cell culture needs to be firmly established. Such information will be important for future pharmacologic and pharmacodynamic studies in developing CO as a therapeutic. To the best of our knowledge, the link between CO and HO-1 induction has never been truly authenticated in a cell culture with rigorous side-by-side comparisons. Third, we note that the reported inference of HO-1 induction effects by exogenous CO in cell culture largely derives from experiments using transition metal-based CORMs.^36−42^ The first reported case of the ability of CO to induce HO-1 expression utilized CORM-2 and CORM-3 and their “CO-depleted” or “inactivated” form (iCORMs) as negative controls in cell culture.^43^ However, the study did not specifically conclude that CO was able to induce HO-1, at least at the concentration of CORMs (less than 100 μM) used in the studies. Nevertheless, “deductive reasoning” and the conventional assumption of CORM-2 and CORM-3 being surrogates of CO might have led to the assumption that CO was the factor that contributed to HO-1 induction, and the conjecture propagates widely in the CO literature. Later studies by other groups using HepG2 cells^44^ and human umbilical vein endothelial cells (HUVEC)^45^ showed the ability of CORM-2 to induce HO-1 over a concentration range of 5–160 μM. Though CO gas was also included in select experiments and showed HO-1 induction activity, the thematic rigor of the studies lies with the work with CORM-2. Fourth, since the initial publications of CORM-2 and CORM-3, it has come to light through rigorous chemistry studies that CORM-2/-3 mostly release CO_2_ instead of CO in the absence of a nucleophile.^46^ Even in cell culture media, they are reported not to release meaningful amounts of CO.^47^ Recently, profound chemical reactivity issues of these ruthenium–carbonyl complexes have been revealed,^20,21,26,28,29,48−51^ including catalase-like activity, reaction with thiol, reduction of Cu(II) to Cu(I), and reduction of NAD(P)^+^ and organic functional groups such as the nitro group. The results from more than 25 publications on CO-independent chemical and biological activity issues have been summarized in a recent review.^29^ Such promiscuous reactivity could lead to general stress and thus the induction of stress response genes (including HO-1) in a CO-independent fashion. To compound all of this, there are no appropriate negative controls available for these Ru-based CORMs. Therefore, whether results generated using these CORMs can be attributed to CO is a fundamental question that needs to be (re)examined. Fifth, the two CORMs frequently used in defining CO biology, including HO-1 induction, are often considered as “positive controls” in the CO field by some. Further, “CO” and “CORM” are quite often used interchangeably in the literature without distinction as if they mean the same thing (they certainly do not). Therefore, there is an elevated sense of urgency to clarify this issue of whether “CO released” from these CORMs induces HO-1 expression in cell culture and by extension, whether these CORMs should be considered as equivalents of “CO.” Lastly, we have developed organic CO prodrugs capable of donating CO under physiological conditions and affording pharmacological effects.^52−54^ These afford us additional tools needed for comparative studies examining the effects of the CO source on HO-1 induction.

For all of these reasons, we became interested in reassessing the reported HO-1 induction effects of “CO” in a few commonly used cell lines including RAW264.7, HeLa, and HepG2 cells. In doing so, we first re-examined the ability of these CORMs to release CO or lack thereof. Then, the effects of CORM-2 and CORM-3 to induce HO-1 in cell culture were examined. We next conducted validation studies under the same conditions using gaseous CO of various concentrations/levels. We also examined organic CO prodrugs BW-CO-103 and BW-CO-111 as CO donors under the same conditions. We also investigated the mechanism of HO-1 induction activity of these agents through the activation of nuclear factor erythroid 2 p45-related factor 2 (Nrf2), an upstream transcription factor of HO-1, and stress-responsive transcription factor.

## Results and Discussion

Before one can attribute the observed biological activity (HO-1 induction activity in the current case) of a chemical CO donor to CO, at least four criteria should be met: (1) the donor should release a sufficient amount of CO under physiological conditions; (2) the donor should have minimal CO-independent activity; (3) the control compounds after CO release should serve as adequate controls for the CO-independent activity of the donor; and (4) CO-induced HO-1 expression can be recapitulated with CO gas. The following discussions are organized according to these criteria.

### (CORM-2/-3–iCORM-2/-3) ≠ CO: CORM-2 and CORM-3 Scarcely Release CO in the Cell Culture Medium of the HO-1 Induction Experiments

**Scheme 1:**
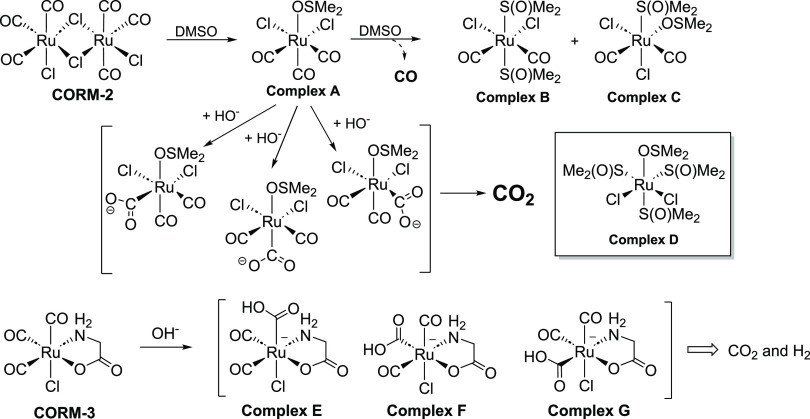
CO Release Chemistry of CORM-2 upon Reacting with DMSO and Water-Gas Shift Reaction of CORM-3 Leading to CO_2_ Generation (Inset Shows the Structure of Complex D Used as iCORM-2 in Some Studies^43^)^21,46,51,60^

**Figure 1:**
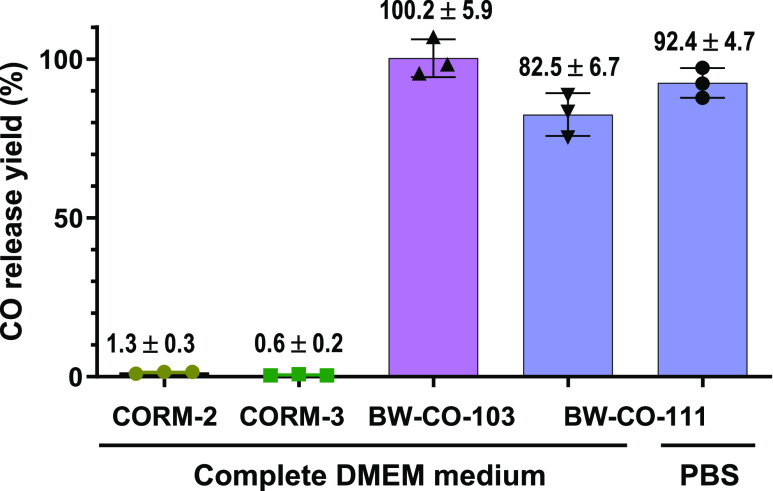
CO release yield of CORMs and CO prodrugs in cell culture medium tested with methanizer-FID GC. Complete DMEM culture medium was supplemented with 10% FBS; concentrations: CORM-2 and CORM-3:50 μM; BW-CO-103 and BW-CO-111:10 μM; incubation time: 6 h. CO release yield was calculated using an external calibration standard curve (Figure S1).

As discussed in the Introduction section, there is a growing volume of evidence of CO-independent activities of Ru-based CORMs including extensive chemical reactivities. Such a CO-independent chemical reactivity and biological activity are not controlled by the commonly used iCORMs. Very critical to the theme of this study, there are extensive literature reports that CORM-2 and CORM-3 do not release meaningful amounts of CO in buffer or in cell culture media.^20,21,46,47,55−58^ As further validation of whether CORM-2 and CORM-3 are able to release CO under our specific experimental conditions, we also studied the release of CO by CORM-2 and CORM-3 after incubation in the cell culture medium in a headspace vial. The CO analytical work was conducted using a methanizer-coupled FID GC, a gold-standard for CO quantitation.^59^ After 6 h of incubation at 37 °C in DMEM culture medium, 50 μM CORM-2 and CORM-3 released 1.3 and 0.6% CO (molar yield based on CO), respectively (Figure 1). It is clear that both CORM-2 and CORM-3 release minimal amounts of CO in the culture medium. Again, such results are consistent with earlier literature reports.^20,21^ As described by Romáo and colleagues, CORM-2 and CORM-3 go through a water-gas shift reaction (Scheme 1) leading to the formation of CO_2_, not CO,^21,46,51,60^ unless in the presence of added nucleophiles such as sodium dithionite.^47,55−57^ In the current study, we also confirmed that both CORM-2 and CORM-3 released less than 2% of CO in the cell culture medium within 6 h. The lack of CO production from CORM-2 or CORM-3 should be sufficient evidence to indicate that the HO-1 induction effect of these two CORMs is not due to CO.

Compared to CORM-2 and CORM-3, the release of CO from BW-CO-103 and BW-CO-111 is almost stoichiometric. A slightly lower yield of CO production was observed for BW-CO-111 than BW-CO-103. This could be attributed to the interference of the serum or thiol species in the culture medium, as the CO release yield is higher in the PBS:DMSO medium. Such results not only confirm the literature conclusions in this regard but also indicate the inadequate nature of CORM-2 and CORM-3 as tools for studying CO biology regardless of whether they induce HO-1 expression. In the end, (CORM-2/-3–iCORM-2/-3) ≠ CO. Further consideration of the chemically reactive nature of CORM-2/-3 and iCORM-2/-3 (as described earlier) leads to the conclusion of an intractably complex situation in terms of what all of these chemical reactions might mean to the subsequent biological response.

### CORM-2 and CORM-3 Induce HO-1 Expression in RAW264.7 and HeLa Cells, But Not Because of CO Release

Next, we attempted to recapitulate the HO-1 induction activity by CORM-2 and CORM-3 so as to establish the basic premise of this study through side-by-side comparisons. In doing so, RAW264.7 and HeLa cells were treated with CORM-2 or CORM-3 for 6 h at two concentrations (50 and 25 μM). Similar concentrations have widely used in literature studies.^36−42^ CDDO-Me was chosen as a positive control due to its known ability to induce HO-1 by activating Nrf2 through binding to Keap1.^61,62^ We opted not to use hemin as a positive control for HO-1 induction because it is also a substrate for HO. We sought to avoid the possible complication due to an increased supply of the substrate for HO, thus increasing the level of CO production.

**Figure 2:**
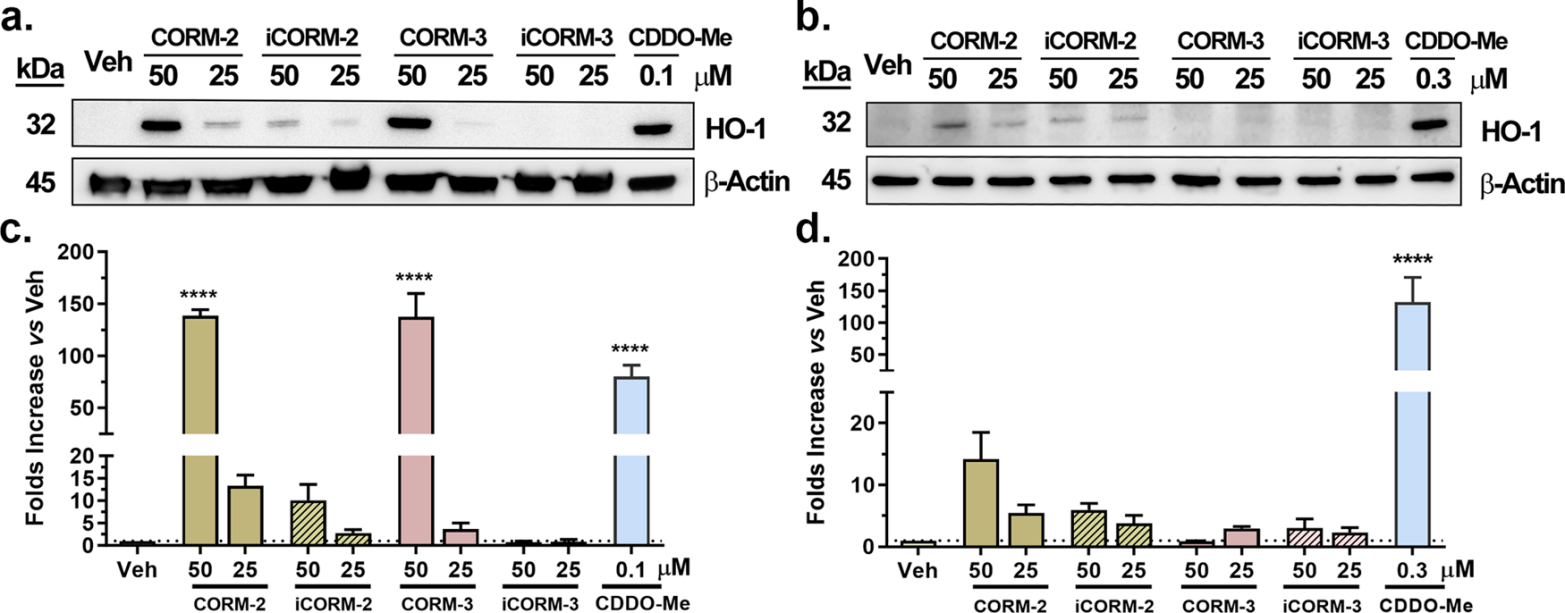
Effects of CORM/iCORM-2 and CORM/iCORM-3 on RAW264.7 cells (a, c) and HeLa cells (b, d). (a) Western blot of RAW264.7 cells incubated with CORM/iCORM-2/-3 for 6 h; (b) Western blot of HeLa cells incubated with CORM/iCORM-2/-3 for 6 h. (c, d) Densitometry results of the Western blot for RAW264.7 cells (c) and HeLa cells (d). (Data show the fold changes compared to the vehicle control group after normalization with band optical density of β-Actin, *n* = 3, ^####^*P* < 0.0001, ^###^*P* < 0.001, ^##^*P* < 0.01, ^#^*P* < 0.05, *t*-test vs Veh group; ****P < 0.0001, one-way ANOVA vs Veh group.).

As shown in Figure 2, CORM-2 significantly induced HO-1 expression in a dose-dependent manner in RAW264.7 cells (Figure 2a,2c). Compared to the vehicle control group (0.5% DMSO), 50 μM CORM-2 increased the HO-1 expression level by about 140-fold. In this case, the “CO-depleted” iCORM-2 also increased HO-1 expression by about 10-fold. As a positive control, CDDO-Me increased HO-1 expression by about 80-fold. In HeLa cells, HO-1 induction by CORM-2 and iCORM-2 was found to be weaker compared to RAW264.7 cells, suggesting a different response to CORM-2 due to differences in cellular phenotypes. Specifically, CORM-2 at 50 μM increased HO-1 expression by about 14-fold (Figure 2b,2d). iCORM-2 also induced HO-1 expression by about 6-fold. Here, we would like to note that, although the bands for HO-1 after CORM induction are clearly visible in the Western blots and one-on-one *t*-test analysis clearly shows statistically significant HO-1 induction in response to CORM-2/iCORM-2 (Figure 2a,2b), the results did not reach statistical significance by one-way ANOVA. Similar to the findings with CORM-2 in RAW264.7 cells, CORM-3 also significantly increased HO-1 expression by approximately 140-fold at 50 μM. However, the induction observed at a lower concentration (25 μM) (Figure 2a,c) only achieved statistical significance by a *t*-test but not by one-way ANOVA. Since 50 μM of CORM-3 is within the range of commonly used concentrations, the results indeed are consistent with literature reports and confirm the HO-1 inducing effects of CORM-3. iCORM-3 under the tested conditions did not induce notable HO-1 expression at the 6-h time point. Further experiments with real-time PCR also confirmed that CORM-2, iCORM-2, and CORM-3, but not iCORM-3 significantly increased the Hmox-1 transcription level in RAW264.7 cells (Figure S3). Overall, the results using CORM-2 and CORM-3 reaffirmed literature findings of their ability to induce HO-1 expression compared to their respective iCORM controls. If one were to believe that the only difference between a CORM and its iCORM is CO and that these two CORMs release sufficient amounts of CO, then it would be reasonable to deduce that the observed difference in their ability to induce HO-1 expression is due to CO. Unfortunately, neither assumption is true. As discussed in the previous section, the lack of CO production from CORM-2 or CORM-3 should be sufficient evidence to indicate that the HO-1 induction effect of these two CORMs is not due to CO. Second, as discussed in the Introduction section, there is a growing volume of evidence of CO-independent activities of Ru-based CORMs including extensive chemical reactivity. Such CO-independent chemical reactivity and biological activity are not controlled by the commonly used iCORMs. Third, there have been extensive studies demonstrating that iCORM-2 and iCORM-3 are not adequate negative controls in terms of controlling for the chemical and biological activities.^29^ The difference in chemical reactivity alone, presumably toward thiols,^20^ between CORM-2 and its controls including CO-depleted iCORM-2 and complex D (Scheme 1) could have accounted for such differences in inducing HO-1 expression.^29^ Overall, these three arguments are supported by extensive published studies, and readers are deferred to our recent review for detailed discussions.^29^ Below, we show that CO gas does not induce HO-1 expression.

### CO Gas Does Not Induce HO-1 Expression in RAW264.7, HeLa, and HepG2 Cell Cultures

As a further step to examine whether CO was the reason for the difference in HO-1 induction ability observed between CORM-2/-3 and iCORM-2/-3, we used CO gas to conduct the same study. The concentration of CO gas was chosen based on literature precedents. Among biological studies using CO gas, 250 ppm CO has been widely used. The associated biological activities include COX inhibition in RAW264.7 cells^63^ and inhibiting LPS-induced expression of Hsp70, HO-1, and Egr-1 in porcine aortic endothelial cells.^64^ CO gas has also been shown to inhibit the proliferation of human airway smooth muscle cells via the Erk1/Erk2MAPK pathway^65^ and migration of various types of cancer cells,^65^ to name a few.^66^ Owing to its tolerance in animal models including rodents, dogs, primates, and humans, 250 ppm CO gas has also been widely used in studying pharmacological activities such as anti-inflammation,^65^ anticancer,^67,68^ and organ protection.^67,68^ Therefore, we chose 250 ppm CO gas as the starting point to probe its effect on HO-1 expression. To be on the safe side in terms of concentration, we also included experiments at 500 ppm, which are higher than what is most widely used in similar experiments.

**Figure 3:**
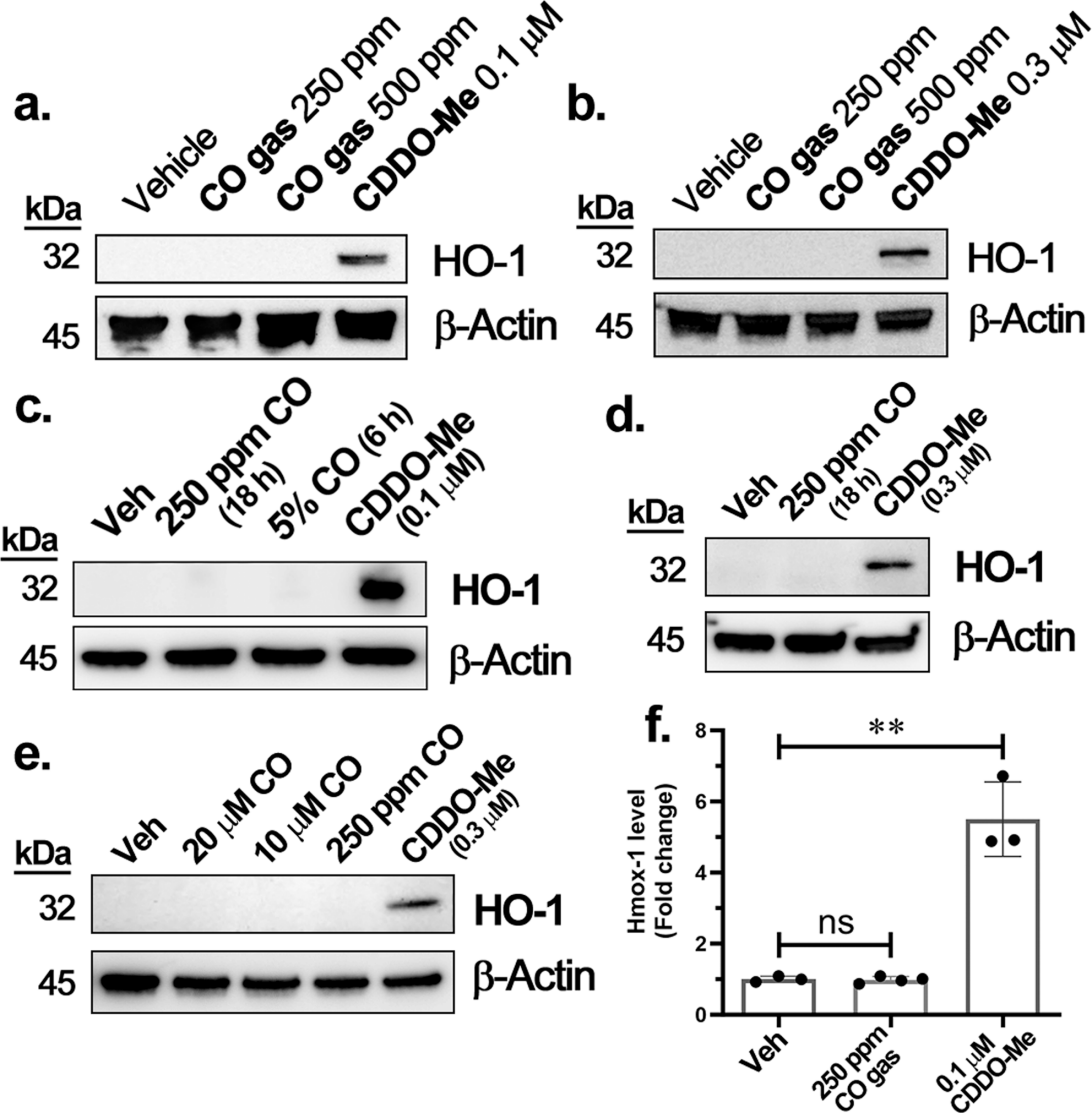
Effect of CO gas on RAW264.7, HeLa, and HepG2 cells: (a) RAW264.7 cells incubated with 250–500 ppm CO gas for 6 h; (b) HeLa cells incubated with 250–500 ppm CO for 6 h; (c) RAW264.7 cells incubated with 250 ppm CO for 18 h or 5% CO for 6 h; (d) HeLa cells incubated with 250 ppm CO for 18 h; (e) HepG2 cells incubated with CO gas dissolved in MEM culture medium (20 and 10 μM) in a normal cell incubator and 250 ppm CO gas in the CO chamber for 6 h; (f) RAW264.7 cells incubated in different conditions (described in the Results and Discussion section) for 6 h. Statistical significance, compared to the vehicle group: *ns:* not significant, **P < 0.01, one-way ANOVA.

HO-1 expression was probed in both RAW264.7 and HeLa cells by Western blot. Incubation of the cells with 250 or 500 ppm CO gas in a gas chamber for 6 h and up to 18 h did not induce any appreciable HO-1 expression compared to the vehicle control (0.5% DMSO). As a positive control, CDDO-Me treatment induced significant HO-1 expression (Figure 3a–c). Similarly, we observed a lack of HO-1 induction by CO in HeLa cells treated with 250 ppm CO gas for 18 h (Figure 3d). Further, to match the similar concentration of the CORMs used (hypothetically assuming CORM-2/-3 release CO), 5% CO gas incubation was also tested (the equivalent of 50,000 ppm), which gives c.a. 50 μM of dissolved CO in the culture medium. Incubation of RAW264.7 cells for 6 h in a gas chamber with 5% CO did not induce any noticeable increase in the level of HO-1 expression (Figure 3c). As further validation experiments, we also studied the effects of CO on HepG2 cells. This was because of a report showing the induction of HO-1 in response to CORM-2 and CO gas in HepG2 cells.^44^ Following the same reported procedure, cells were incubated with 10–20 μM CO solution, 250 ppm CO gas, or CDDO-Me as the positive control. Neither CO solution nor CO gas induced notable HO-1 expression in HepG2 cells (Figure 3e). Moreover, we also used rRT-PCR to evaluate the Hmox-1 mRNA level in RAW264.7 cells. Hmox-1 transcription was induced after the addition of CDDO-Me (0.1 μM) or hemin (50 μM) to the RAW264.7 cells. Consistent with the results of Western blot, incubation of RAW264.7 cells with 250 ppm CO gas for 6 h did not increase Hmox-1 transcription compared to the vehicle control (0.5% DMSO) (Figure 3f). To be on the safe side in terms of concentration, we also incubated RAW264.7 cells with 5% CO (50,000 ppm) and did not observe induction of Hmox-1 transcription when compared to the control (Figure S2).

### Effect of a Representative Organic CO Prodrugs on HO-1 Expression

**Figure 4:**
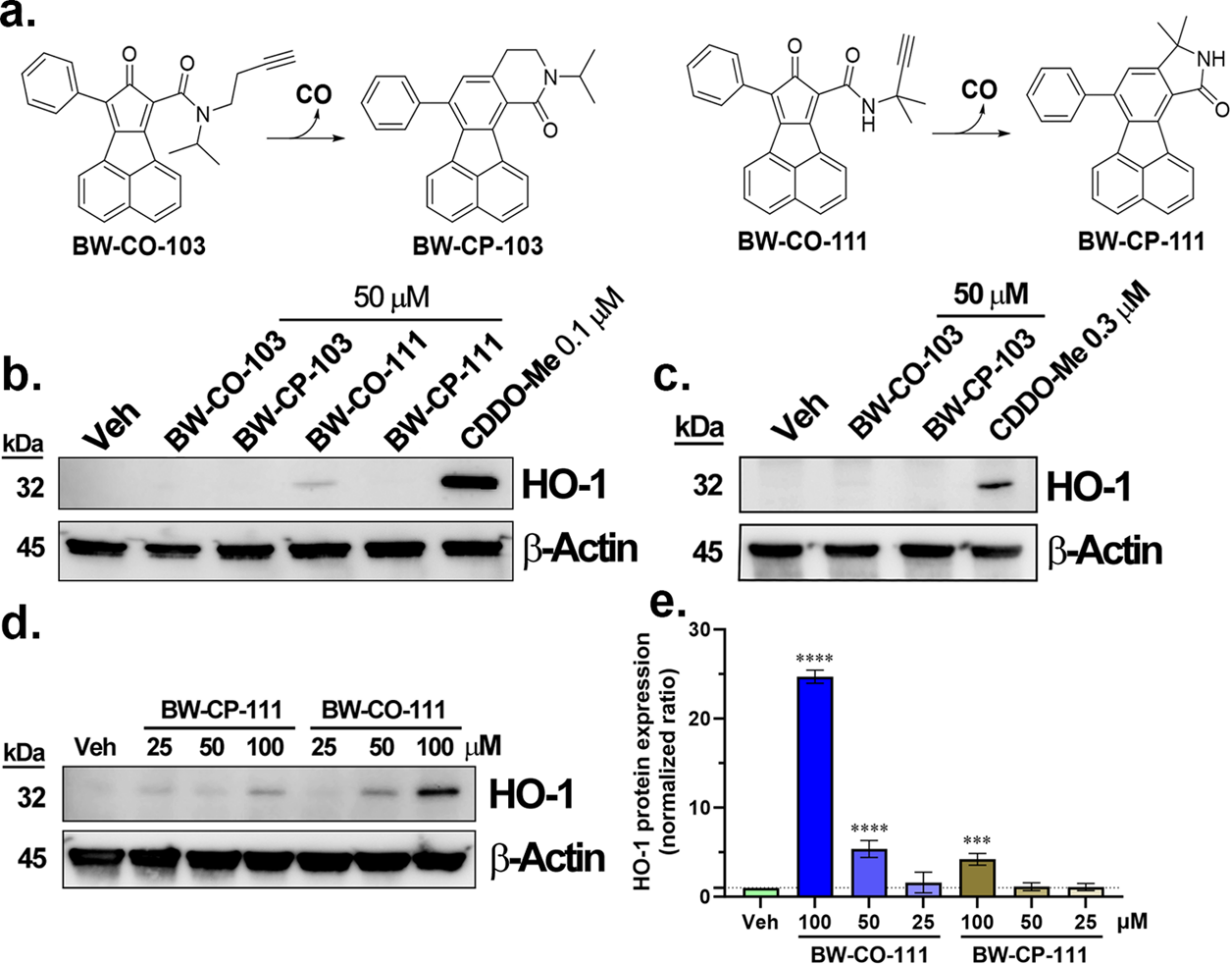
Effect of CO prodrugs on RAW264.7 and HeLa cells. (a) CO release chemistry of BW-CO-103 and BW-CO-111; (b) Western blot of RAW264.7 cells incubated with BW-CO/CP-103 or BW-CO/CP-111 for 6 h; (c) Western blot of HeLa cells incubated with BW-CO/CP-103 for 6 h. (d) Dose-dependence test of HO-1 induction activity of BW-CO/CP-111 in RAW264.7 cells at 6 h time point; (e) densitometry analysis of the Western blot of BW-CO-111 (Data show the folds change comparing to the vehicle control group (0.5% DMSO) after normalization with the optical density of the β-Actin band (*n* = 3, ****P < 0.0001, ***P < 0.001 vs Veh group, one-way ANOVA)).

It is important to mention that one active area of research in studying the therapeutic effect of CO is the development of noninhalation CO delivery forms, including carbonyl complexes with transition metals and borane,^69−73^ CO in solution,^74^ and photosensitive transition metal complexes and organic CO donors.^75−79^ In 2014, we introduced the first organic CO prodrugs capable of donating CO under physiological conditions and affording pharmacological effects.^52−54^ We have shown that these CO prodrugs release almost stoichiometric amounts of CO under physiological conditions. Therefore, we selected two CO prodrugs, BW-CO-103^54^ and BW-CO-111.^80^ Their CO release mechanisms are shown in Figure 4a. The CO release half-lives were found to be 1.3 h and 14 min for BW-CO-103 and BW-CO-111 in aqueous solution, respectively. As shown in Figure 1, we also verified their sufficient CO release yield in the cell culture medium used for the following Western-blot studies (Figure 4) and rRT-PCR (Figure S3). Cell uptake of BW-CO-103/-111 in RAW264.7 and HeLa cells were verified by monitoring the formation of the fluorescent CO-depleted products, BW-CP-103/-111, by fluorescence microscopy (Figure S4). In Western-blot studies, incubation of BW-CO-103 and BW-CP-103 for 6 h did not induce notable HO-1 expression in either RAW264.7 or HeLa cells (Figure 4b,c). However, at 50 μM, BW-CO-111 induced HO-1 expression in RAW264.7 cells by about 4-fold, similar to that observed with 25 μM CORM-3. To further confirm the HO-1 induction activity of BW-CO-111, a dose–response study was conducted. It was found that at 100 μM, both BW-CO-111 and BW-CP-111 induced HO-1 expression to a significant level of about 25-fold and 4-fold higher than the background level, respectively (Figure 4d,e). On the other hand, in rRT-PCR studies in RAW264.7 cells, Hmox-1 transcription levels were significantly increased by the treatment with 50 μM BW-CO-111 and BW-CO-103 compared to the vehicle control and 250 ppm CO gas groups. It is conceivable that 50 μM CO prodrugs could deliver more CO into the cells than 250 ppm CO gas; we also tried incubating RAW264.7 cells with 5% CO gas for 6 h, which should give 50 μM CO in the medium by calculation. However, the Hmox-1 level remained unchanged (Figure S2). Such results indicate that the need to examine the CO-independent effects of a CO donor is not limited to metal-based CORMs. It is critical that attention be paid to potential CO-independent activities of chemical CO donors in studying CO biology in cell cultures or animal models.

It needs to be noted that inhaled CO in animal models and healthy human subjects has been shown to induce tissue HO-1 expression in the liver (mice),^81,82^ cardiac muscle (rats),^83^ and skeletal muscle (human).^83^ Especially significant is a human-subject study in 37 healthy volunteers. The group receiving 200 ppm CO (1 h a day) for 5 days showed a 2–3-fold increase in the HO-1 level in the biopsy samples of vastus lateralis muscle compared to the control group receiving air.^83^ The results we obtained from cell culture studies do not directly extrapolate to in vivo systems, as one would expect a living organism to have much more complex responses toward CO, including secondary, tertiary, and even synergistic responses. Indeed, such a discrepancy argues for the need for more studies to understand the mechanism(s) responsible for the functions of the CO–HO-1 axis in the body.

### Nrf2 Induction Activity of CO Gas and CO Donors

**Figure 5:**
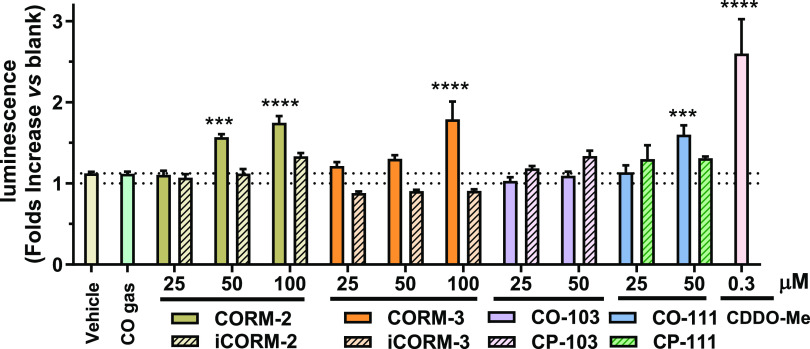
Nrf2 activation level tested with the Nrf2/ARE-luciferase reporter assay in transgenic HEK293 cells (0.5% DMSO in cell culture medium was used as the vehicle for all groups); CO gas concentration was 250 ppm, *n* = 3, data show the adjusted ratio by dividing chemiluminescent signal of the tested sample with the chemiluminescent signal of the naive Nrf2-luciferase cells (Compared to the vehicle group: ***P < 0.001, ****P < 0.0001, one-way ANOVA).

In order to further probe the mechanism of action for these CO donors to induce HO-1, we looked into the Nrf2/HO-1 pathway. Nrf2 is a key transcription factor that functions to regulate antioxidant and detoxification gene expression to maintain cellular homeostasis. Under cellular stress conditions, Nrf2 is stabilized through modification of its negative regulator Kelch-like ECH-associated protein 1 (Keap1) through the reaction with electrophiles or oxidants. Such stabilization leads to Nrf2 accumulation in the nucleus to initiate the expression of cytoprotective genes through binding to ARE in their respective promoters.^84^ CORM-2 and CORM-3 have been reported to react with thiol groups; such reactions contribute to their CO-independent biological activity including cellular stress.^36^ Since HO-1 is one of the key downstream target genes of Nrf2, CORM-2 and CORM-3 may induce HO-1 through activation of Nrf2 as reported in previous studies.^36,45,85^ To examine this and also gain some mechanistic insights into whether CO gas can activate Nrf2, a HEK293 cell line with a stably transfected Nrf2/ARE-luciferase reporter was treated with CO gas, CO donors, and their corresponding controls.^86,87^ As shown in Figure 5, treating the reporter cells with 0.3 μM CDDO-Me for 6 h as a positive control significantly increased the Nrf2 transcriptional activity. Such results are consistent with literature precedents that CDDO-Me activates Nrf2 through binding with Keap1.^61^ In line with the Western-blot results, 250 ppm CO gas did not change Nrf2 transcriptional activity compared to the vehicle control group. For treatments with CORMs, 100 and 50 μM CORM-2 and 100 μM CORM-3 significantly increased transactivation of Nrf2. Western-blot studies also verified that nuclear Nrf2 of HeLa cells was significantly increased by treatment with 100 μM CORM-2 compared to the vehicle control but not by 250 ppm CO gas (Figure S5). Interestingly, in Nrf2 reporter cells, iCORM-3 marginally decreased the luminescence intensity compared to the vehicle and naïve control groups, although the change was statistically insignificant. For the organic CO prodrugs, BW-CO/CP-103 did not significantly alter the activity of Nrf2, consistent with Western-blot results. However, 50 μM BW-CO-111 significantly activated the Nrf2/ARE reporter, in line with the observed induction of HO-1 expression. Such results are consistent with the HO-1 expression level assessment and suggest that HO-1 activation by CORM-2/-3 and BW-CO-111 is likely due, at least in part, to activation of the Nrf2/Keap1/ARE pathway, possibly via reacting with the thiol species.^20,48,88,89^ One potential noteworthy aspect aside from the Nrf2 pathway is the effect of CO toward Bach1, a heme-responsive HO-1 transactivation repressor.^90^ Heme induces expression of Hmox-1 in part by inhibiting the binding of Bach1 to the Hmox-1 enhancers and inducing the nuclear export of Bach1.^91^ Although CO alone does not seem to directly affect Hmox-1 transcription, it will be worthwhile to further study if the CO donors in question can activate HO-1 through inhibiting Bach1 as seen with cadmium and ASP8731.^92,93^ It would be even more intriguing to see, as a product of heme catabolism, whether CO could affect HO-1 expression in the presence of heme. More work is needed to understand related mechanistic questions, especially in the context of *in vivo* actions, which offer more interesting factors.

## Conclusions

In conclusion, our studies show that CO gas at 250, 500, or 5% (50,000 ppm) was unable to induce HO-1 expression in RAW264.7, HeLa, and HepG2 cells. Exposure to a 10–20 μM CO solution for 6 h also had no noticeable effect on HO-1 expression in HepG2 cells. In contrast, some CO donors and their control compounds, including CORM-2, iCORM-2, and CORM-3 were found to induce HO-1 in RAW264.7 and HeLa cells at concentrations that impart biological effects.^43,94^ Importantly, CORM-2 and CORM-3 only release a scarce amount of CO. These results clearly indicate that the HO-1 induction effects of CORM-2 and CORM-3 were not due to CO. The results further suggest the need to apply stringent criteria in attributing the observed effects from these (and any) CO donors to CO. It is also important to comprehensively consider the chemical reactivity and potential interaction of the CO donors/prodrugs toward biological entities such as cofactors,^95^ amino acids,^20^ proteins,^21^ nucleic acids,^96^ and even the biological assay reagents^26^ before attributing the observed biological activities to CO. This is especially important if the donor has known and significant chemical reactivity. Such is the case with carbonyl–ruthenium complexes. It is generally accepted that nothing is truly benign without considering the context of concentration. This point is especially true when a transition metal complex is administered into a biological system. Further, there is no absolutely perfect negative control compound for a donor/prodrug molecule for CO-independent effects arising from the chemical donor itself. This is because the donor and control compound are indeed chemically different entities. With these considerations in mind, it is very important to use more than one source of CO in studying its biology. Further, well-defined CO release chemistry, stoichiometry, and kinetics represent a minimal requirement for a CO donor to be used for studying CO biology. We also note that the different results from the different cell lines studied have significant mechanistic implications and suggest differential effects of CO among different cell types. Therefore, an inference on the applicability of our findings (the lack of HO-1 induction by CO) in other cell lines can only be made after actual experimental verifications. Further, these findings cannot be directly extrapolated to animal models for obvious reasons including the heightened complexity of the whole organism, the effect of general stress, and the enhanced possibility of indirect and/or synergistic effects. Much more work is needed. However, one thing is clear: induction of HO-1 by CORM-2 and CORM-3 in RAW264.7, HeLa, and HepG2 cells is not attributable to the “CO released” from these CORMs as commonly suggested.

## Material and Methods

### Materials

#### Compounds and Stock Solutions

CORM-2 and CORM-3 were purchased from Sigma-Aldrich and used without further characterization. iCORM-2 and iCORM-3 were prepared by incubating the 10 mM stock solution of CORM-2 in DMSO or 10 mM stock solution of CORM-3 in DMEM culture medium (without FBS) at 37 °C overnight, followed by purging with nitrogen for 20 min. Freshly made stock solutions (10 mM) of CORM-2 or CORM-3 were made with DMSO and nanopure water, respectively, preceding the addition to the cell culture wells. BW-CO/CP-103 and BW-CO/CP-111 were prepared routinely in our lab according to the published procedures.^54^ Stock solutions of these compounds were made in DMSO (20 mM) preceding the addition to the cell culture wells. DMSO was kept at 0.5% in all compound treatment groups and in the vehicle control group.

### Gas Chromatography

CO release of the CO donor was tested with an Agilent (Santa Clara, CA) 7820A system equipped with a purged packed inlet (operates at 150 °C), Restek 5A mole sieve column (2m, 0.53 mm ID, helium carrier gas at 4.5 mL/min flow rate), Restek (Bellefonte, PA) CH4izer methanizer coupled with an FID detector (methanizer H_2_ flow rate: 25 mL/min, FID H_2_ flow rate: 15 mL/min, air flow rate: 400 mL/min, methanizer temperature: 380 °C, FID temperature: 300 °C), oven temperature program: 0–4 min 100 °C then increase to 250 °C at a rate of 60 °C/min and hold at 250 °C for 4 min followed by a decrease to 100 °C at a rate of 60 °C/min then hold at 100 °C for 2 min. The typical CO peak is eluted at 1.3–1.4 min. To test the CO release yield, the stock solution of CORMs or CO prodrugs prepared as indicated in the previous section was added to the 2 mL headspace vial containing 1.5 mL complete cell culture medium or PBS (headspace volume: 0.5 mL), and the vail was instantly sealed with an aluminum cap with silicone-PTFE septum. For CORM-2 and -3 and BW-CO-103, DMSO concentration was 0.5%. The headspace vial was then incubated in a temperature-controlled shaker at 37 °C for 6 h. 100 μL headspace gas was then injected into GC. CO concentration in the headspace was quantified using an external standard curve established by injecting 100 μL of certificated CO calibration gas (10, 20, 50, 100, 10,000 ppm) purchased from GASCO (Oldsmar, FL). CO release yield was calculated by
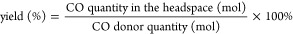


### Cell Culture

RAW264.7 cells, HeLa cells, and HepG2 cells were purchased from the American Type Culture Collection (ATCC, Manassas, VA). RAW264.7 cells were cultured in Dulbecco’s modified Eagle’s medium (DMEM, Corning) supplemented with 10% fetus bovine serum (FBS, Corning) and 1% penicillin/streptomycin (PNS). HeLa cells were cultured in DMEM (without pyruvate) containing 10% FBS and 1% PNS. HepG2 cells were cultured in Minimum Essential Medium Eagle (MEM, Corning) medium supplemented with 10% FBS and 1% PNS. Cells were seeded in a 6-well plate and treated after confluency reached about 80–100%. CO gas treatment was conducted under 37 °C with a gastight chamber (2500 mL volume, Mitsubishi Gas Chemical, Japan) equipped with needle valve gas inlet and outlet ports under constant supplement (5 mL/min) of premixed CO gas (250 ppm CO, 5% CO_2_ in balanced air, Airgas, PA). 5% CO gas treatment was achieved by equilibrating the cells in the gas chamber with the aforementioned 250 ppm premixed CO gas together with a balloon containing 125 mL of pure CO gas (Airgas, PA) sealed by a magnetic clamp. After equilibration, the inlet and outlet valve were closed and the clamp was removed by magnet force applied outside of the chamber, leading to the release of the packed CO into the chamber to achieve the designated concentration. CO solution treatment was conducted according to a reported procedure.^44^ Briefly, the HEPES buffer was saturated by bubbling pure CO gas for 20 min. The CO saturated buffer (c.a. 1 mM CO) was further diluted to the designated concentration by mixing with the MEM complete culture medium preceding addition to the cell culture wells.

### Western Blot

Primary monoclonal antibodies (mouse origin) for HO-1 (sc-390991) and β-actin (sc-8432) were purchased from Santa Cruz Biotechnology (Texas). The secondary antibody (goat-antimouse HRP-conjugated IgG) was purchased from Biorad (Hercules, CA). After treating the cells with specified conditions, the culture medium was aspirated, and the cells were washed three times with PBS. Cells were lysed with 120 μL of 1× Laemmli loading buffer (contains 2.5% mercaptoethanol) and denatured at 95 °C for 5 min. 2–5 μL of denatured cell lysate was loaded onto the 10% SDS-PAGE gel (Biorad), and electrophoresis was conducted with a Biorad system under constant voltage (150 V). The protein was transferred to the PVDF membrane with a Trans-Blot Turbo RTA transfer kit (Biorad). The membrane was blotted by the iBind Flex system (Thermo Fisher), using HO-1 (1:100), β-actin (1:400), and HRP-conjugated secondary antibody (1:1500). The chemiluminescence of the protein band was imaged with LAS4000 (GE Healthcare, Chicago, IL) and analyzed with InteliQuant software.

### Real-Time PCR

After treating RAW264.7 cells with specified conditions, total RNA was isolated by using an RNeasy Mini Kit (Qiagen, Germany). The Taqman Gene Expression Assays (Applied Biosystems, Foster City, CA) used are as follows: β-actin (assay ID: Mm00607939_s1) and Hmox-1 (assay ID: Mm00516005_m1). rRT-PCR was performed using TaqPath 1-step Multiplex Master Mix (Applied Biosystems) on Illumina Eco Real-Time PCR System (San Diego, CA). β-actin was used as the reference gene. Data were analyzed by the comparative *C*_T_ method (ΔΔ*C*_t_) using Eco Real-Time PCR software.

### Nrf2/ARE-Luciferase Reporter Assay

Nrf2/ARE-luciferase reporter stable HEK293 cell line was purchased from Signosis (Santa Clara, CA). The cell line has been stably transfected with the pTA-ARE-luciferase reporter vector, which contains four repeats of antioxidant response elements (ARE) of the promoter upstream of the firefly luciferase coding region along with a hygromycin-resistant vector. Cells were cultured and tested according to the manufacturer’s manual. Briefly, the stable reporter cell line was cultured in DMEM culture medium containing 10% FBS, 1% PNS, and 50 μg/mL hygromycin B (Sigma-Aldrich, St. Louis, MO) in humidified air with 5% CO_2_ at 37 °C. When reached 90% confluency, cells were trypsinized and seeded onto a white clear-bottom 96-well plate (Corning, Corning, MA) at a density of 2 × 10^4^ cells/100 μL/well and cultured overnight. Cells were treated with various conditions for the designated time, washed with PBS before lysing with lysis buffer (Signosis), and incubated with luciferase substrate solution (Signosis). The chemiluminescent signal was read by a PerkinElmer Vector 3 plate reader (Waltham, MA). After subtraction with the signal of the blank wells, the chemiluminescence signal of all treatment groups was normalized by the signal of the nontreatment group (naive reporter cells).

### Statistical Analysis

Results are expressed as the mean ± SD. Comparison of the means of two groups was done by the *t*-test; multiple group comparisons were done by one-way ANOVA using GraphPad Prism 9.0. The statistically significant level was denoted in the corresponding figures and the figure legends, where *P* < 0.05 was considered significant.

## Supporting Information Available

Supplemental experimental methods and additional figures including CO quantification, rRT-PCR results, cell imaging, and Western blot of nuclear Nrf2 (PDF)
