# Supplementary material for: On the Question of CO’s Ability to Induce HO-1 Expression in Cell Culture: A Comparative Study Using Different CO Sources
Source: ACS Chem Biol. 2024 Feb 10;19(3):725–35. doi: 10.1021/acschembio.3c00750 (PMC10949199; doi:10.1021/acschembio.3c00750)
Supplement: Supplementary file 1 — cb3c00750_si_001.pdf [file cb3c00750_si_001.pdf]

Supporting Information for

On the Question of CO's Ability to Induce HO-1 Expression in Cell Culture: A Comparative Study Using Different CO Sources

Xiaoxiao Yang, Qiyue Mao, and Binghe Wang*

Department of Chemistry and Center for Diagnostics and Therapeutics, Georgia State University, Atlanta, Georgia 30303, USA

***Correspondence:** wang@gsu.edu

Table of Contents

Page No.

Supplemental Figures (S1-S5)……………………………..……………………………..…. S2-S4

Supplemental Experimental Methods ………………………….……..…………….S4-S5

Supplemental Figures

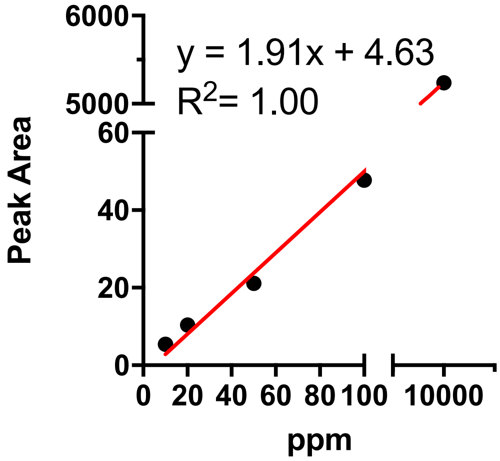

Figure S1. An external standard curve used for quantifying the CO concentration in headspace vials.

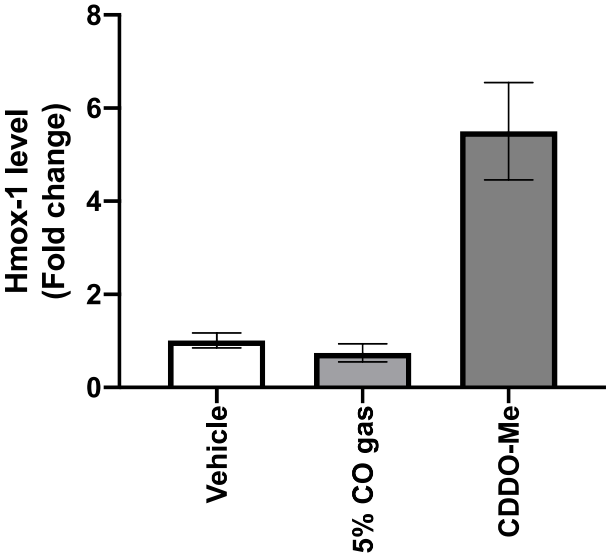

Figure S2. Hmox-1 transcription levels of RAW264.7 cells incubated with 5% CO gas, CDDO-Me (0.1 μM) for 6 h.

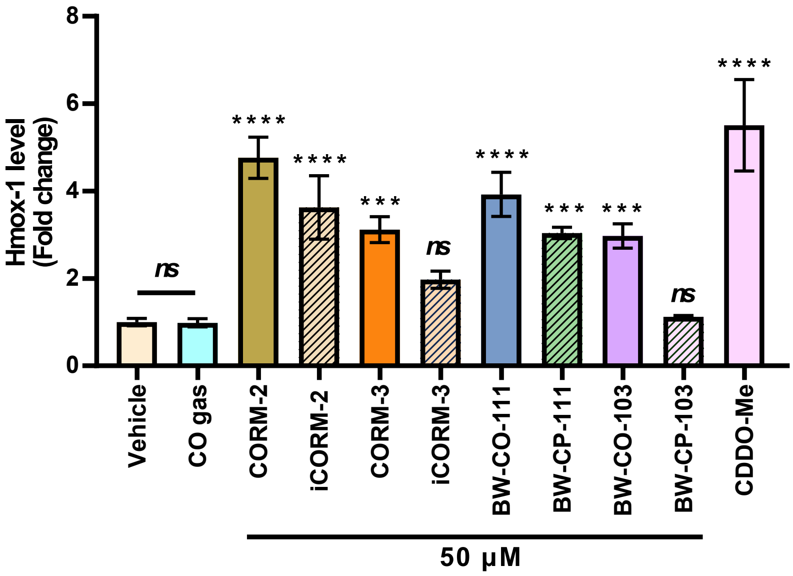

Figure S3. RAW264.7 cells incubated with different compounds for 6 h (detail is described in the experiment section). Concentrations: CO gas: 250 ppm, CDDO-Me: 0.1 μM, other compounds were incubated at 50 μM. Statistical significance, compared to the Vehicle group: ns: not significant, ***P<0.001, ****P<0.0001, one-way ANOVA.

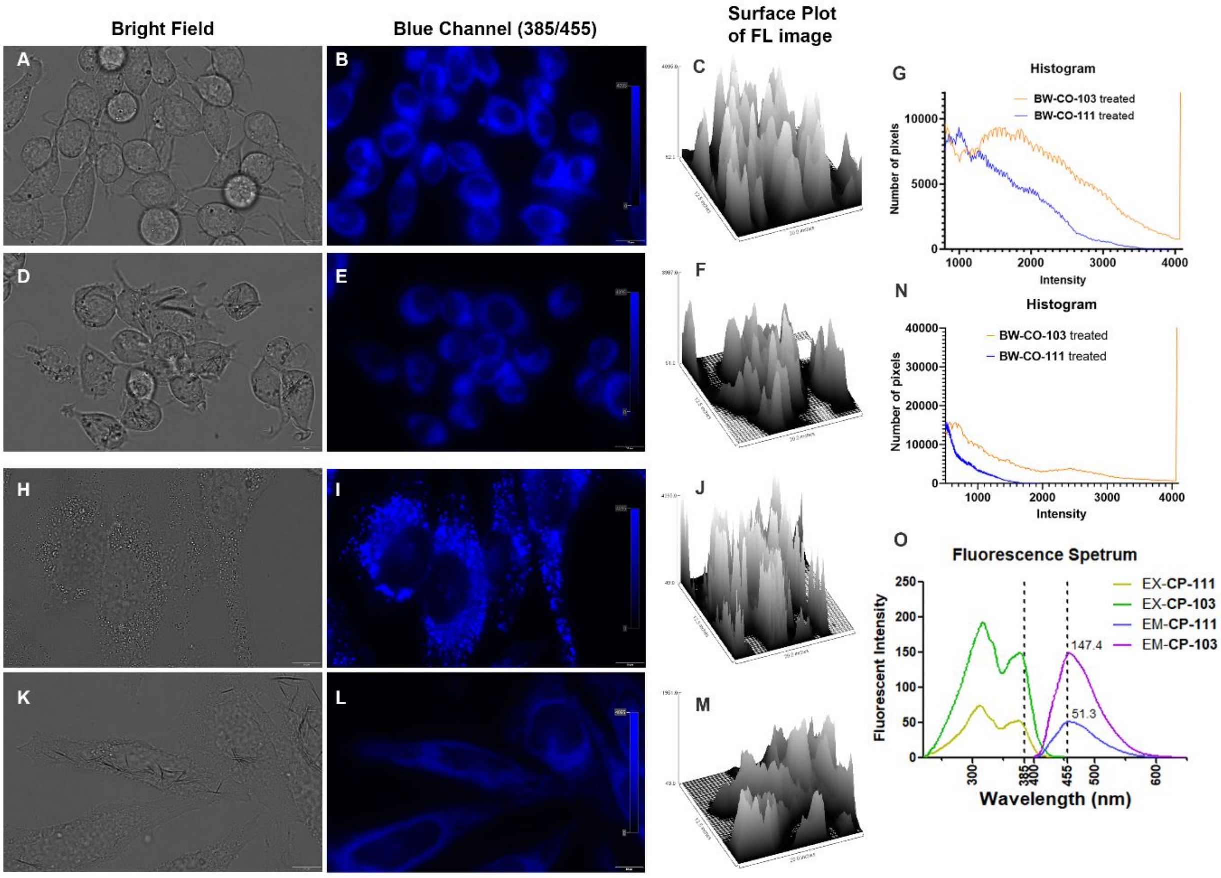

Figure S4. Cell imaging results showing internalization of **BW-CO-103** and **BW-CO-111** in

RAW264.7 and HeLa cells. (A-C) Bright field image, blue channel image, and surface plot of blue fluorescence channel image of RAW264.7 cells treated with 50 μM **BW-CO-103**; (D-F) bright field image, blue channel image, and surface plot of blue fluorescence channel image of RAW264.7 cells treated with 50 μM **BW-CO-111**; (G) comparison of the histograms of B and E; (H-J) Bright field image, blue channel image, and surface plot of blue fluorescence channel image of HeLa cells treated with 50 μM **BW-CO-103**; (K-M) bright field image, blue channel image, and surface plot of blue fluorescence channel image of HeLa cells treated with 50 μM **BW-CO-111**; (N) comparison of the histograms of I and L; (O) fluorescent excitation (Em: 377 nm) and emission (Ex: 460 nm) spectra of BW-CP-103 and BW-CP-111, dashed lines mark the wavelength used by the fluorescence microscope (bandwidth: 5 nm). scale bar: 10 μm, low intensity portion of the histogram was omitted for clarity.

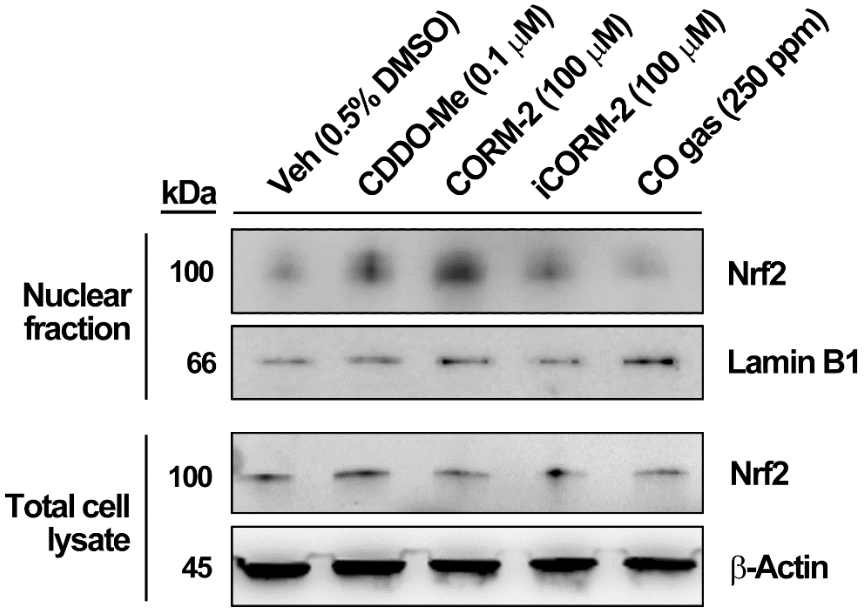

Figure S5. Wester-blot showed significant increases in Nrf2 expression in the nuclear fraction of HeLa cells treated with 100 μM CORM-2 and iCORM-2 for 2 h. 250 ppm CO gas treatment for 2 h did not increase nuclear Nrf2 level. CDDO-Me (0.1 μM) was used as the positive control. Lamin B1 and β-Actin were used as the loading control for nuclear fraction and total cell lysates, respectively.

<u>Supplemental Experimental Methods</u>

Cell Imaging

RAW264.7 and HeLa cells were seeded in the 3.5 cm Cellvis glass bottom culture dish (Mountain View, California, USA) at a density of 1×10^4/dish in DMEM medium (Corning, Corning, New York, USA) supplemented with 10% FBS, 100 units penicillin, and 100 μg/ml streptomycin. After culturing overnight, the cells were treated with 50 μM **BW-CO-103** or **BW-CO-111** for 6 h. Cells were then washed twice with PBS and incubated in fresh FluoroBrite DMEM medium (ThermoFisher, Carlsbad, California, USA). The live cells were imaged with Olympus IX-73 inverted fluorescence microscope using 100x oil immersion lens under blue channel and phase-contrast transillumination settings. Fluorescent images were taken under the same exposure settings including excitation light intensity and exposure time. Raw images were processed with ImageJ software.

Wester-blot for Nuclear Nrf2

Hela cells (3×10^6 cells) were culture in 10 cm Petri dish overnight in 5% CO_2 humidified cell culture incubator. After treatment with specified conditions: Vehicle control: 0.5% DMSO, 100 μM CORM-2/iCORM-2, 0.1 μM CDDO-Me, and 250 ppm CO gas for 2 h, cells were washed with cold PBS for 2 times. The nuclear fractions were separated by using the pre-extraction buffer (supplemented with DTT and protease inhibitors) provided in the Nuclear Extraction Kit (ab113474, Abcam, Waltham, MA, USA) following the instruction provided by the kit. The nuclear fractions were lysed with 1× Laemmli loading buffer containing 2.5% 2-mercaptoethanol. The total cell lysate was prepared by lysing the treated cells with 1× Laemmli loading buffer containing 2.5% 2-mercaptoethanol. The heat-denatured samples were used for Western-blot studies as described in the Experimental section of the main manuscript. Primary antibodies: Nrf2 (D1Z9C, Rabbit mAb #12721, Cell Signaling Technology, 1:1000), Lamin B1 (D9V6H, Rabbit mAb #13435, Cell Signaling Technology, 1:1000). Secondary antibody: Immun-Star GOAT Anti-Rabbit (GAR)-HRP Conjugate (Bio-rad, #170-5046, 1:20000).
